# MR‐ARFI to evaluate intensity variability and enhance targeting of FUS: A brain study in rats

**DOI:** 10.1002/mrm.70039

**Published:** 2025-09-02

**Authors:** Abubakr Eldirdiri, Linda Chang, David A. Martin, Eric Cunningham, Segun Bernard, Donna J. Calu, Kim Butts Pauly, Thomas Ernst

**Affiliations:** ^1^ Department of Diagnostic Radiology and Nuclear Medicine University of Maryland School of Medicine Baltimore Maryland USA; ^2^ Department of Neurobiology University of Maryland School of Medicine Baltimore Maryland USA; ^3^ Department of Radiology Stanford University Stanford California USA

**Keywords:** low‐intensity focused ultrasound (LIFU), magnetic resonance acoustic radiation force imaging (MR‐ARFI), neuromodulation, targeting accuracy, ultrasound pressure variability

## Abstract

**Purpose:**

Inconsistencies in focused ultrasound (FUS) transducer positioning and skull‐induced aberrations can reduce the targeting accuracy and cause inconsistencies in the intensity delivered during FUS neuromodulation procedures. This study aimed to evaluate the use of MR‐acoustic radiation force imaging (MR‐ARFI) in improving the targeting accuracy and assessing the variation in the pressure delivered during FUS procedures.

**Methods:**

An MR‐guided FUS system was used to bilaterally target the nucleus accumbens region of Sprague–Dawley rats. Displacement maps were acquired with MR‐ARFI to refine the targeting accuracy by adjusting the focal point position electronically and mechanically as necessary. In addition, the ARFI measurements were used to establish the relationship between the displacement and input power, and to assess intra‐ and inter‐subject variability in FUS pressure.

**Results:**

ARFI displacement was a strong predictor of FUS power (*r* ^2^ = 0.935, *p* < 0.001), with displacements showing quadratic dependence on FUS pressure. Intra‐site and intra‐subject coefficients of variation in ARFI displacement measurements were 9.0% and 18.6%, whereas the variation across animals was 43.4%. Initial MR‐guided targeting required secondary adjustments in 21% of cases.

**Conclusion:**

Despite initial MRI guidance, substantial targeting errors were observed, highlighting the need for advanced imaging techniques like MR‐ARFI in neuromodulation procedures. Moreover, ARFI displacements in the target region across subjects varied over three‐fold, indicating very high variation in the delivered acoustic pressure. By improving the precision of FUS targeting and estimating deviations in the FUS pressure, MR‐ARFI can improve therapeutic outcomes and reduce risks in transcranial FUS neuromodulation.

## 1 INTRODUCTION

Low‐intensity focused ultrasound (LIFU) is an emerging promising technique for neuromodulation.^1^, ^2^, ^3^ Unlike deep brain stimulation and optogenetic interventions which are invasive and carry a higher risk of serious adverse events,^4^, ^5^ LIFU offers a non‐invasive approach to effectively target specific brain regions. Other non‐invasive techniques such transcranial magnetic stimulation and transcranial direct current stimulation suffer from limited depth penetration and poor spatial resolution.^6^, ^7^ Initial studies have shown great potential of LIFU for treating various neurological disorders, including essential tremors^8^ and substance use disorders,^9^, ^10^ as well as reduction of temporal summation of pain.^11^

However, skull‐induced focused ultrasound (FUS) aberrations, as well as inconsistencies in FUS transducer positioning and electronic steering, can cause distortion in the FUS beam shape and deviations in the location of the focal point from the intended target. These effects may also lead to inaccuracies in the energy deposition at the targeted region, thus reducing the efficacy of the neuromodulation treatment and increasing the risk of side effects.^12^, ^13^ Numerical simulations informed by CT or MRI skull data have been proposed to estimate the spatial distribution of the FUS intensity field,^14^, ^15^, ^16^ but these imaging data and processing pipelines are not readily available and can be costly. MR guidance can improve the accuracy in LIFU treatments. For instance, MR visible markers or radiofrequency trackers can be used to identify the FUS transducers and estimate the focal point relative to the brain.^17^ However, even these improved techniques may suffer from experimental errors such as skull aberrations and poor acoustic coupling between the transducer and the skull.

MR acoustic radiation force imaging (MR‐ARFI) can locate the focal spot and correct for acoustic aberrations during FUS therapy.^18^ MR‐ARFI works by encoding the small displacements induced by the FUS waves during sonication, using strong bipolar magnetic field gradients. The measured displacement at the focal spot is typically in the order of micrometers. Numerous studies have been conducted to optimize MR‐ARFI in terms of SNR^19^, ^20^, ^21^, ^22^ and acquisition speed.^23^, ^24^, ^25^ MR‐ARFI has been proposed as a method to iteratively correct transcranial phase aberration^26^, ^27^ and to assess the variation in tissue properties during FUS procedures.^28^, ^29^ A recent study also showed that MR‐ARFI can be used in combination with MR elastography to estimate the intensity of FUS in phantoms with a range of viscoelastic properties.^30^

Since FUS neuromodulation parameters that may affect treatment outcomes, such as delivered pressure and localization, can be largely unknown and difficult to control across subjects, we propose the use of MR‐ARFI to improve the targeting accuracy and to reduce variations in ultrasound (US) dose in brain FUS procedures. Similarly, correlations between ARFI displacement and neuromodulation efficacy was demonstrated in large animal models,^31^ but a systematic evaluation of MR‐ARFI's reproducibility and inter‐/intra‐subject variability remains lacking. This study addresses that gap by evaluating these factors in a small animal model. The work presented here was conducted in preparation for a LIFU study targeting the nucleus accumbens (NAc), the reward center, to reduce drug seeking behaviors or drug use in rats trained to self‐administer fentanyl. We demonstrate the added benefit of using MR‐ARFI in refining the targeting accuracy over an MR‐gFUS system that relied solely on external markers (US transducer) for localization. Additionally, we used ARFI to estimate the intra and inter‐subject variabilities in FUS pressure delivered to target brain regions.

## 2 METHODS

All experiments were conducted on a 7T small animal MRI scanner (BioSpec 70/30 Avance III, Bruker, Ettlingen, Germany), using a transmit‐receive single‐loop radiofrequency coil (40 mm × 35 mm). FUS sonication was performed using an MR‐compatible FUS system (Image Guided Therapy (IGT), Pessac, France) equipped with eight transducer elements operating at 1.5 MHz. The focal region of the FUS system had an ellipsoidal shape, with an estimated radial diameter of 1 mm and an axial length of 10 mm, based on simulations conducted in a prior study using the same transducer configuration.^32^ The FUS platform utilizes a 2D positioning mechanism to move the transducer in the horizontal plane above the skull and allows electronic adjustment of position along the beam axis.

**FIGURE 1:**
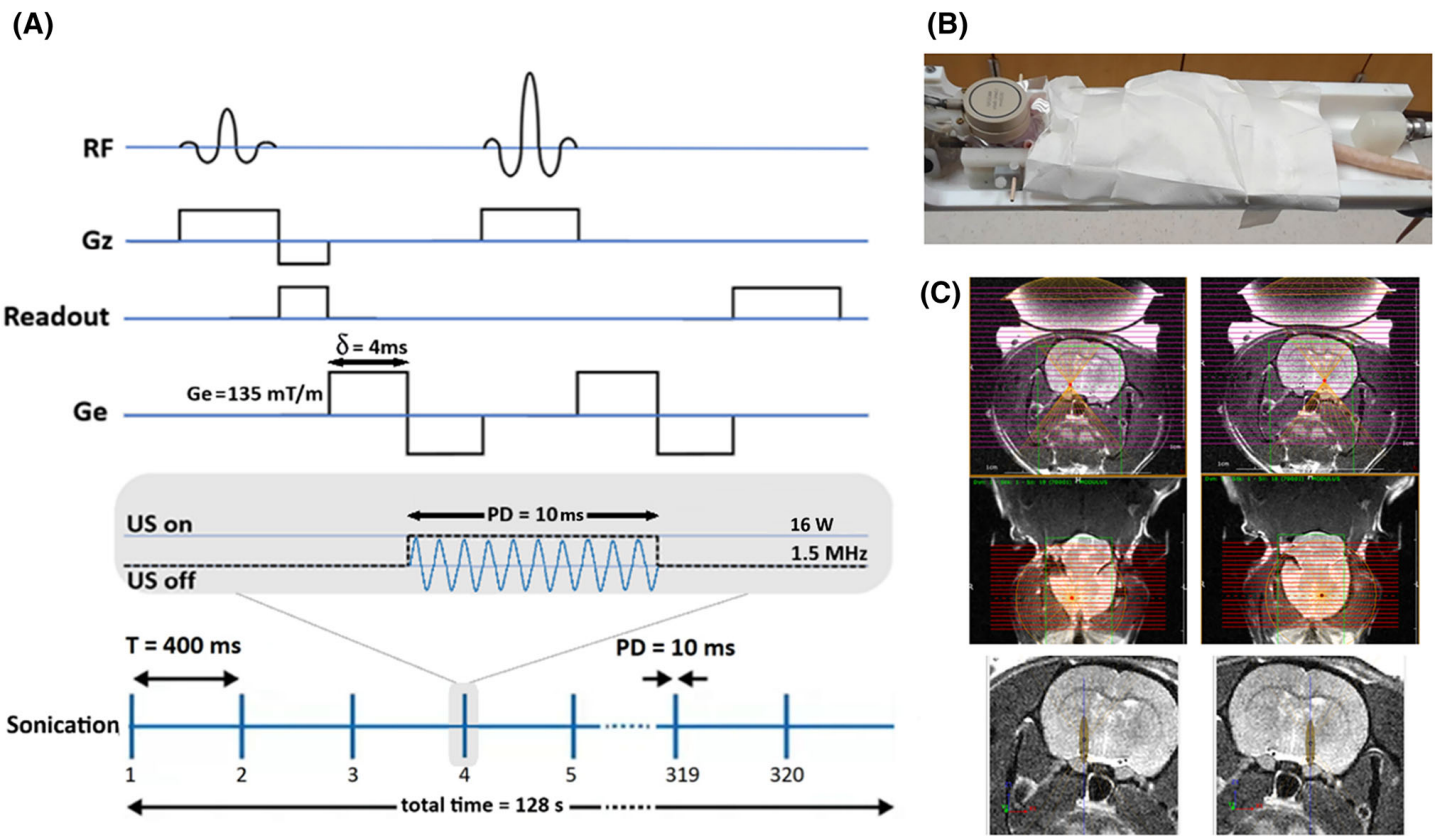
(A) The spin echo ARFI sequence and sonication scheme. (B) FUS device placed on top of the rat's head. (C) The adjustment of the focal point location using the IGT software. The IGT mechanical system allows two‐degree horizontal (in plane) movement of the transducer on top of the skull. The depth (along the beam) is controlled electronically.

For ARFI, five slices approximately perpendicular to the FUS beam direction were acquired with the following imaging parameters: spin‐echo with TE 25 ms, TR 2000 ms, FOV 50 × 50 mm^2^, matrix size 64 × 64, and slice thickness 2 mm with no gap. The bipolar motion encoding gradients had a strength of 135 mT/m and a duration of 2 × 4 ms. Throughout the ARFI animal experiments, the US pulse duration (PD) was fixed at 10 ms and synchronized with the two central lobes within the bipolar motion encoding gradients (see Figure 1A). A fixed pulse repetition period of 400 ms was used, resulting in a duty cycle of 2.5%. A total of 320 pulses were delivered per ARFI acquisition. These parameters were consistent across all experiments. The FUS power settings used in the different experimental groups are summarized in Table 1. The five ARFI slices were acquired twice, once with FUS turned on and once with FUS turned off.

**TABLE 1:** Summary of the animal experimental groups and the FUS power settings used.

Experiment	Purpose	No. of animals	Status	Sites targeted	Input power	Pulses	Repeats
1	Assess ARFI displacement vs. input power; measure temperature at max power	1	Anesthetized	Right NAc	9.6–22.4 W	320	1 per power setting (total: 7)
2	Assess intra‐site, intra‐subject, and inter‐subject variability	5	Euthanized	Left and Right NAc	16 W	320	5 per site (10 per animal)
3	Assess inter‐subject variability and ARFI‐guided targeting	21	Anesthetized	Left and Right NAc	16 W	320	1 per site (2 per animal)

**FIGURE 2:**
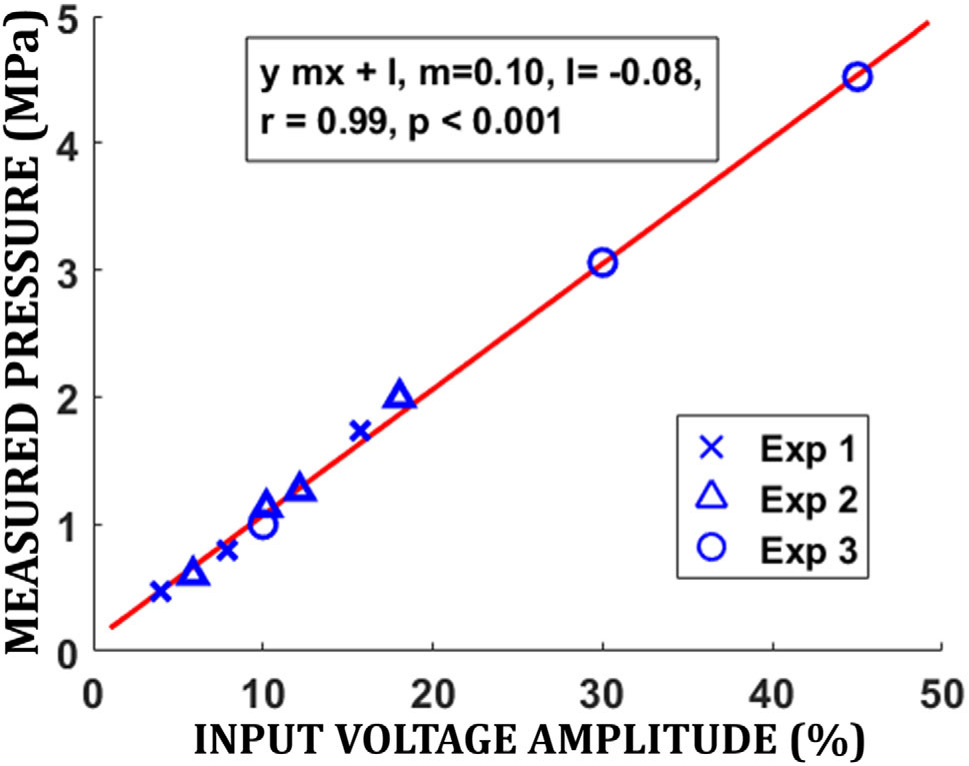
Linear relationship between the amplitude of the input voltage (percentage with respect to a maximum amplitude of 40 V) and the pressure at the focal point obtained by calibrating the FUS device using a hydrophone. Calibration data were acquired in 3 separate sessions, indicated by crosses (experiment 1), triangles (experiment 2), and circles (experiment 3).

All in vivo animal experiments were performed in accordance with National Institutes of Health (NIH) guidelines for animal welfare using protocols approved by the University of Maryland Baltimore Institutional Animal Care and Use Committee. In all animal experiments, the NAc region was targeted bilaterally in the brain of Sprague–Dawley rats. These animals weighted between 215 g and 565 g. Three types of MR‐guided FUS animal experiments were performed. The purpose, FUS parameters and number of animals used in these experiments are summarized in Table 1. The first experiment was conducted to establish the relationship between the ARFI displacement at the focal point and the input power. In this experiment, multiple ARFI acquisitions were performed with the FUS targeting the right NAc in the brain of one anesthetized rat and with power level varying from 9.6 W to 22.4 W (30%–70% of maximum power). This power range corresponds to pressure values ranging from 5.0 MPa to 8.2 MPa, based on the calibration described below. In this experiment, MR thermometry was used to measure the temperature rise at the focal region with the highest power setting (22.4 W). The second experiment was conducted on five rats that were euthanized for another study to investigate the intra‐subject variability in the measured ARFI displacement. In each animal, the focal point of FUS beam was moved to target the left and right NAc and 5 measurements of ARFI displacement were made at each site with input power of 16 W (50% of maximum power), which corresponds to 7 MPa in the US gel as per calibration equation from Figure 2. The third experiment was conducted in 21 anesthetized animals to determine the variability of ARFI displacement measurement and presumably FUS pressure across animals as well as the variability in ARFI displacements between left and right targeted NAc regions. Again, the input power was set to 16 W and the focal point was moved to the left and right NAc in each animal. In this experiment one ARFI displacement measurement was acquired per site. This third set of experiments was also used to assess the utility of MR ARFI to improve the FUS targeting as described below.

To monitor the temperature at the focal point during ARFI acquisitions, MR thermometry based on the proton resonance frequency shift was performed. This sequence was used to monitor temperature rise during sonication with the same FUS settings as in the ARFI experiment with the highest input power of 22.4 W. This sonication scheme consisted of 320 pulses with a period of 400 ms and a duration of 10 ms per pulse, for a total sonication of 128 s. The MR thermometry sequence was a gradient echo sequence with the following parameters: TE 8 ms, TR 15 ms, FOV 40 × 40 mm^2^, matrix size 96 × 96, slice thickness 2 mm, 120 acquisitions, and total acquisition duration 173 s. The slice positioning was based on the ARFI acquisition to ensure inclusion of the focal point.

### 2.1 FUS calibrations

The FUS system was first calibrated to verify the relationship between the applied voltage (in percent of maximum voltage) and the pressure at focus. This calibration was conducted outside the MRI scanner in US gel (Aquasonic® 100, Parker Laboratories Inc., NJ 07004, United States) using a calibrated hydrophone (sensitivity (at 1.5 MHz) = −242.5 db re. 1 V/1uPa, HNR‐1000 Onda Corporation, Sunnyvale, CA, US) and an oscilloscope. To ensure the hydrophone was at the FUS focal point, the hydrophone was initially moved in the US field until the maximum peak voltage was detected. The input electric power to the FUS device was then gradually varied and the voltage amplitude measured with the oscilloscope at the focal point was recorded and converted into pressure. To ensure that the applied US dose is accurately controlled and consistent across the different FUS sessions, three independent calibration experiments were performed on different days.

### 2.2 MR‐guided FUS procedure to target the NAc

During MRI‐guided FUS, rats were anesthetized with 1.5%–2.0% vaporized isoflurane and 100% oxygen (flow rate = 1 L/h) administered through a nosecone. Physiological parameters were monitored using an SAII MRI‐compatible unit (Small Animal Instruments Inc., Stony Brook, NY). The hair on the animal head was shaved before applying US gel and placing the transducer on top of the head, Figure 1B.

Following positioning of the animal and FUS system at the magnet isocenter, an initial adjustment of the FUS transducer position was made by acquiring a T_2_‐weighted spin echo sequence with TR 3000 ms, TE 36 ms, field of view 40 mm × 40 mm, matrix size 128 × 128, slice thickness 1 mm without gap, and a total of 30 slices. The IGT software package was then used to predict the location of the FUS focal point relative to the brain, and the mechanical and electronic shifts needed to move the focal point to the left or right NAc were applied (Figure 1C).

Next, the actual FUS focus of the initial MR‐guided alignment was measured using ARFI and the error relative to the target region (e.g., left of right NAc) was determined. If the initial focal point was incorrect, the targeting of the FUS system was refined by adjusting the focal point either electronically along the beam direction or radially by mechanically moving the FUS transducer horizontally above the head. The ARFI acquisition was repeated to ensure that the NAc region was accurately targeted.

### 2.3 Image analysis

ARFI displacement maps were generated as follows. First, the brain was segmented by thresholding the magnitude images, resulting in a brain mask. Next, the phase of the ARFI images was unwrapped within the brain mask. The reference phase, obtained from the set of slices with sonication turned off, was then subtracted from the ARFI phase images to eliminate phase variations due to magnetic field inhomogeneities and eddy currents. Finally, the phase difference images were converted to displacements d using the formula:

d=∆Φ/(γGT)

where ∆Φ is the phase difference from ARFI, γ is the proton gyromagnetic ratio, G is the amplitude of the encoding gradients, and T the total duration of two gradient lobes.

The temperature rise was estimated from the series of the phase images obtained with the MR thermometry acquisition. The phase in these images was unwrapped and the initial phase image, $\Phi \left(t_{0}\right),$ was subtracted from the phase image, Φ(t), at each time point, t, in the series. The phase difference images, $\Phi \left(t\right)-\Phi \left(t_{0}\right),$ were converted to a temperature change by

$$∆T\left(t\right)=\frac{\Phi \left(t\right)-\Phi \left(t_{0}\right)}{\gamma \alpha B_{0}\;\mathrm{TE}}$$

where γ is the gyromagnetic ratio, α is the proton resonance frequency change coefficient of −0.01 ppm/°C, $B_{0}$ is the magnetic field strength, and TE is the echo time.

To evaluate the viability of the measured displacement maps at varying FUS power levels in experimental group 1, we calculated the SNR map at each power level. The SNR at power level i was computed as the point‐wise ratio between the ARFI displacement map and the standard deviation (SD) of the noise in the ARFI measurement:

$${\mathrm{SNR}}_{i}\left(x,y\right)=\frac{d_{i}\left(x,y\right)}{\sigma_{0}}$$

where $d_{i}\left(x,y\right)$ is the displacement at power level i, and $\sigma_{0}$ is the SD of the displacement map acquired at zero acoustic power (i.e., without sonication).

## 3 RESULTS

### 3.1 Calibration experiments

The outcome of the FUS calibration is shown in Figure 2. The amplitude of the electric voltage applied closely predicted the pressure measured with the hydrophone (*r* ^2^ = 0.994) across three independent calibration experiments (triangles, crosses, and circles in Figure 2). Hence, the applied US dose is accurately controlled and consistent across different FUS sessions.

### 3.2 Estimating pressure variations using ARFI

**FIGURE 3:**
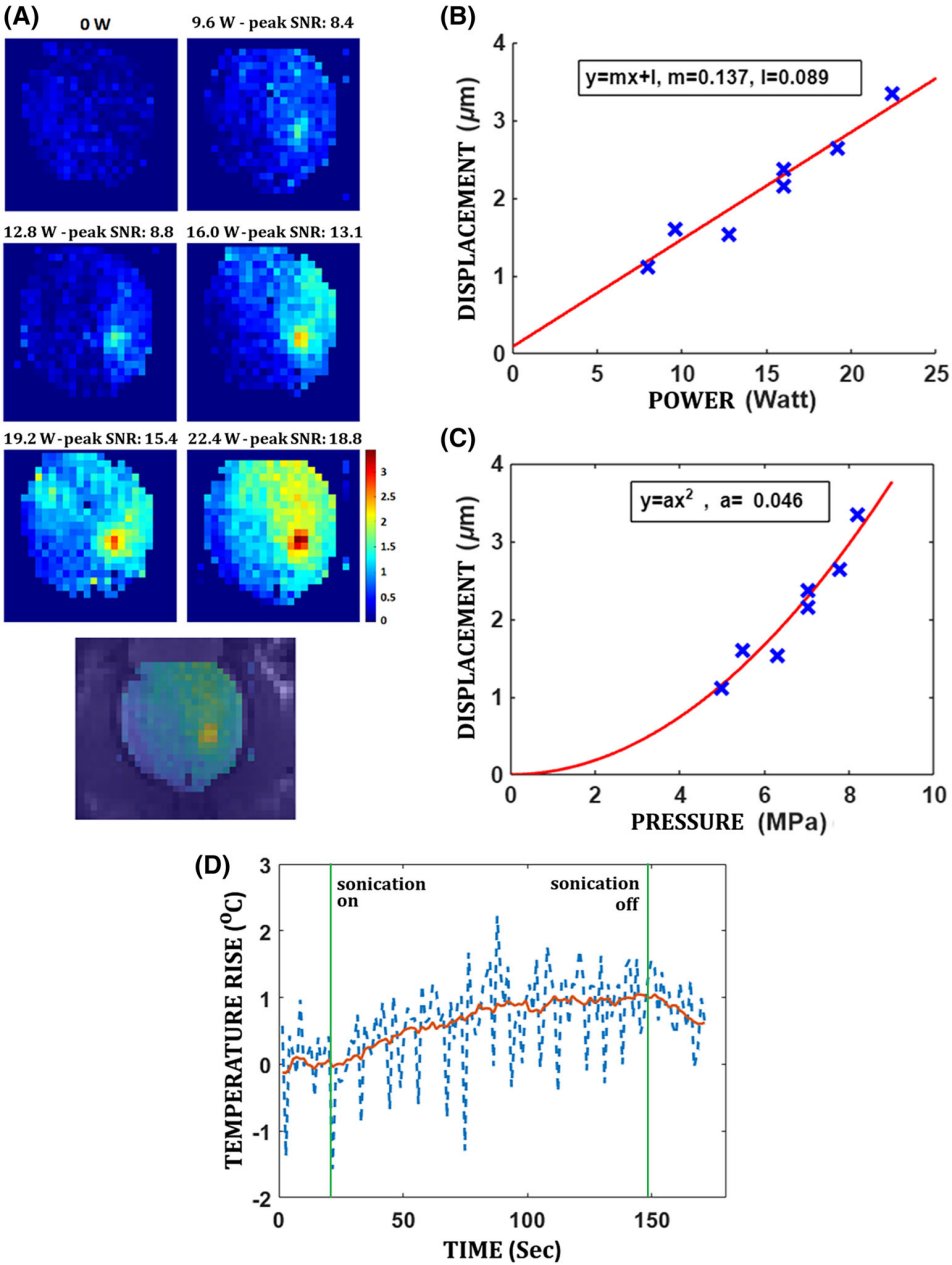
(A) ARFI displacement maps obtained for different input power levels on anesthetized rat. The SNR reported in (A) represents the peak value at the focal point. The noise SD was calculated from the displacement map obtained with a power setting of 0 W. (B, C) Relationship between the displacement measured at the focal point and the input power and pressure in a single animal experiment. (D) Raw and smoothed temperature rise (in blue and red) in the brain, from a region of 7 × 7 pixels (or 3 × 3 mm^2^) encompassing the focal point, during ARFI sonication scheme with power of 22.4 W. The green lines mark the start and the end of the sonication.

Figure 3 illustrates the tissue displacements measured on an anesthetized rat with ARFI at different FUS power levels. Within the range of experimental parameters, the displacement from ARFI showed a close linear relationship with the FUS power (linear regression: *r* ^2^ = 0.935, *p* < 0.001). Likewise, ARFI displacement showed a quadratic dependence on FUS pressure estimated at the focal point. Importantly, the observed brain temperature increase recorded on MR thermometry with 22.4 W power setting remained within 1°C, Figure 3D. Moreover, an improved SNR was observed in the phase‐encoded displacement measured at the focal point as the applied FUS power increased.

**FIGURE 4:**
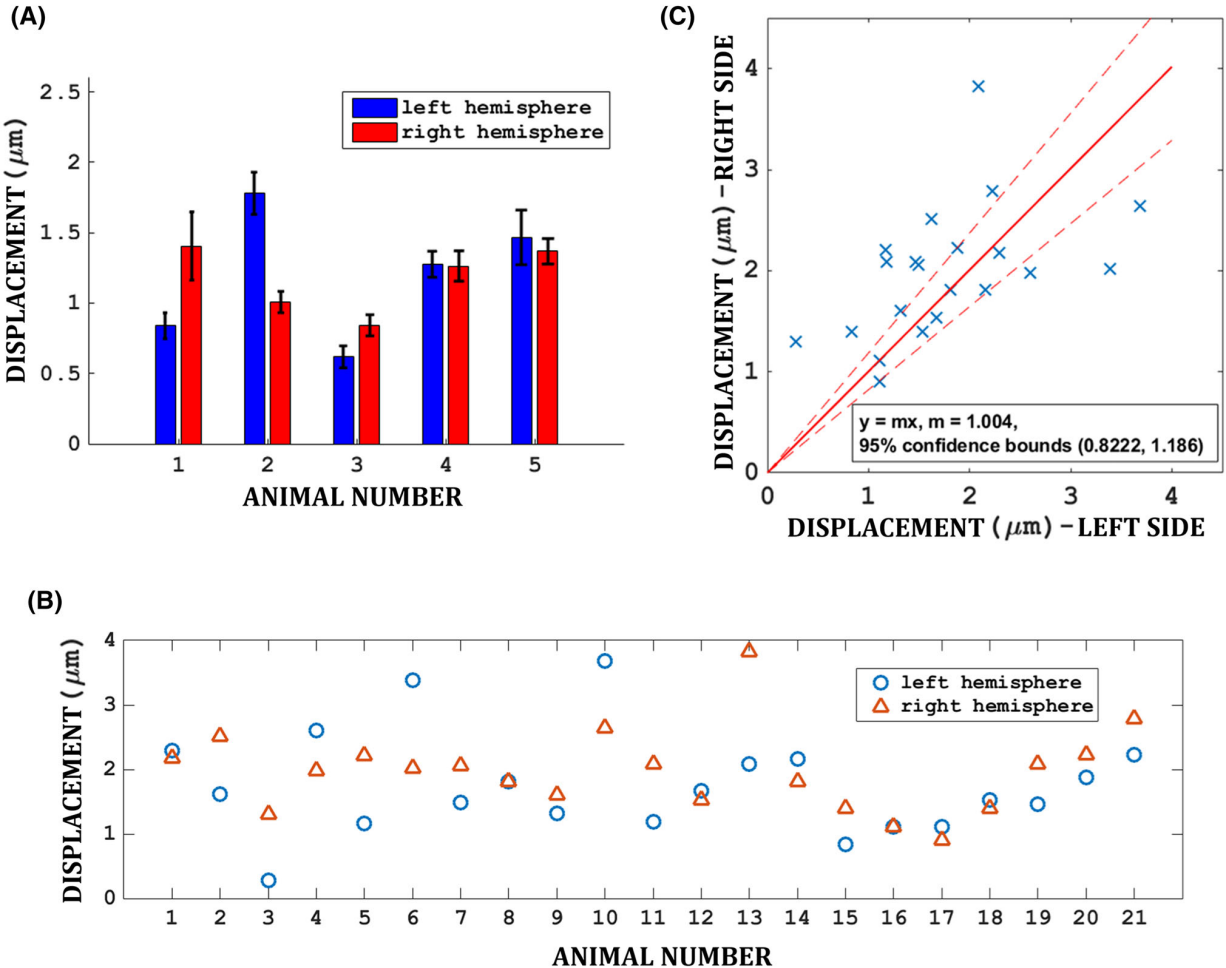
(A) Reproducibility of ARFI displacement in 5 animals, each measured five times, at the left and right NAc regions. Error bars indicate the SD of repeated measurements at the same site without moving the transducer. (B) The ARFI displacements measured at left and right NAc across 21 animals are shown. (C) Correlation between the measured ARFI displacement at the left and right NAc.

Figure 4A shows the within‐subject variability of ARFI displacements in the left and right hemisphere of five animals. The intra‐site coefficient of variation (CoV) of the ARFI displacements, defined as the SD of repeated measurements at the same site without moving the transducer divided by its mean, was 9.0% ± 3.0% (mean ± SD). Conversely, the intra‐subject CoV (across left and right hemispheres in each animal) was 18.6% ± 10.2%, and the CoV across all five subjects was 30.2%.

Figure 4B shows the ARFI displacements in 21 animals at the left and right NAc regions. Although the same power of 16 W, corresponding to 7 MPa in the US gel phantom (and approximately 4 MPa in situ^12^), was applied by the FUS system, the ARFI‐encoded displacement in the NAc had high variability (CoV) of ±43.3% across experiments (1.80 ± 0.78 μm). Because of the close correlation between ARFI displacements and acoustic pressure (Figure 3), these findings suggest a similarly large variability in the delivered energy to the target region across animal experiments. The ARFI displacement was not significantly different between hemispheres (difference 0.20 μm; *p* = 0.20, paired t‐test) but the ARFI displacements in the left and right NAc regions were correlated (Figure 4C). The intra‐subject CoV across the left and right hemispheres was 23.4% ± 20.5%.

### 3.3 Localization errors

Subsequent to the initial MR‐guided targeting, MR‐ARFI further improved the localization in almost one quarter (10 of the 42) of the experiments. Initial localization errors were predominant (9/10) along the beam's long axis (depth); hence, we were able to adjust the focal point location electronically. One case demonstrated an initial error in the focal point perpendicular to the beam axis, which was resolved by mechanically moving the FUS transducer horizontally above the skull.

**FIGURE 5:**
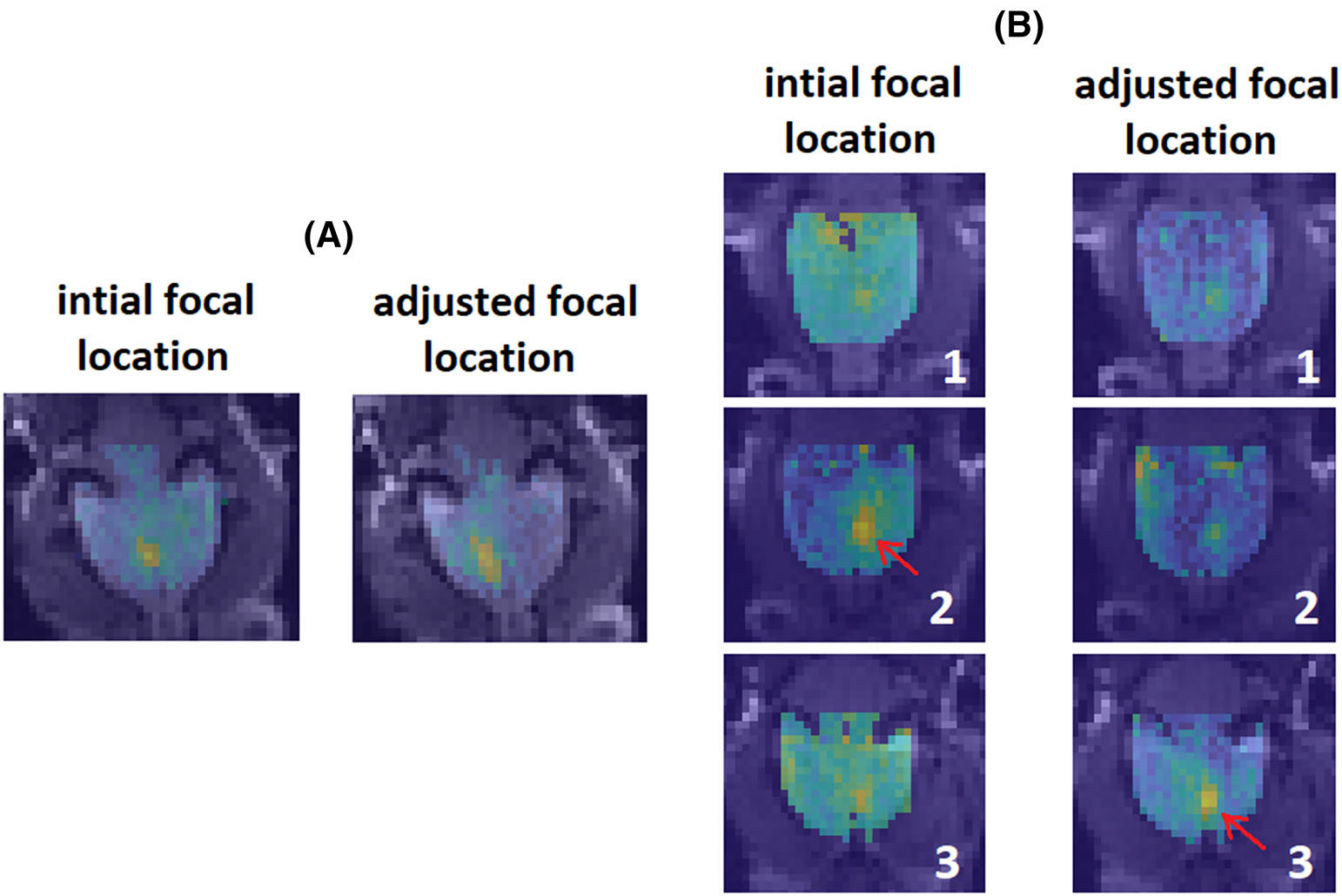
Improved NAc targeting based on ARFI images. (A) The target had to be shifted in‐plane by 1.5 mm away from the midline. (B) The target needed to be shifted through‐plane by 2 mm (from slice 2 to slice 3, red arrows).

In MRI coordinates, the initial focal point was adjusted 2.7 mm on average for the nine cases that required electronic adjustment (along the beam axis) and 1.5 mm for the case that required mechanical adjustment. Figure 5 illustrates instances where the initial targeting was incorrect and how ARFI improved the targeting. In Figure 5A the initial focus was located too close to the center of the brain; accordingly, the FUS transducer was moved mechanically by 1.5 mm to the left. Figure 5B shows ARFI displacement maps from three consecutive brain slices in one animal. Initially, the maximum ARFI displacement was in slice number 2 (left column, red arrow), above the location of interest. Hence, the FUS trajectory was adjusted electronically to lower the focus by one slice (2 mm; right column, red arrow).

## 4 DISCUSSION

Our work demonstrates the usefulness of MR‐ARFI to assess the variability in FUS pressure delivered during FUS treatment. Moreover, we show that MR‐ARFI is a valuable tool to reduce the targeting error and improve the accuracy in MRI‐guided FUS procedure. The observed discrepancies in targeting, despite initial MRI guidance, and the variation in FUS energy delivered to the targeted region highlight the challenges posed for FUS techniques that do not use MR‐ARFI. The errors and variability observed may cause suboptimal therapeutic outcomes in treatment studies, potentially diminishing the efficacy of neuromodulation and increasing the risk of targeting error that might lead to adverse effects.

Our results align with a prior study that demonstrated the utility of MR‐ARFI displacements measurements in a large animal model to confirm the targeting accuracy and showed that the ARFI displacements correlated with neuromodulatory effects, specifically with the suppression of visual evoked potentials.^31^ In addition, our study aimed at evaluating intra‐ and inter‐subject variability of MR‐ARFI displacements across a larger cohort of small animals. Together, these studies underscore the importance of MR‐ARFI in improving the outcome of FUS neuromodulation and facilitating its translation to clinical practice.

Our calibration experiments demonstrate a close linear relationship between the FUS input electric voltage and the pressure at the focal point l (*r* ^2^ = 0.994). This consistency across calibration sessions underscores the reliability of our FUS system in delivering a controlled US dose and ensures that the variation in the FUS intensity from hardware setting is minimized. Note that, since the calibration was performed in US gel, the pressure values reported here are expected to be slightly lower than those measured in a water field. The attenuation coefficient for US gel is 0.09 db/cm/MHz compared to 0.02 db/cm/MHz for water. A limitation in the calibration experiment was that the hydrophone used (with a 2.5 mm active element diameter) is relatively large compared to the small lateral focal width of the transducer. This likely led to a slight underestimation of the peak pressure due to spatial averaging. A more accurate pressure estimate could be obtained using needle hydrophones with smaller active element diameters. Another limitation was that the upper limit of our calibration apparatus prevented direct measurement of pressure at the input power level used for ARFI (16 W or ˜7 MPa), requiring extrapolation to estimate the pressure at that point; this introduces potential uncertainty due to possible nonlinearity in the pressure–voltage relationship at high input levels.

We observed an improved SNR in the phase‐encoded displacement measured with ARFI as the applied FUS power increased. To minimize the applied power while maintaining sufficient SNR, we selected a power level of 16 W for subsequent experiments aimed at enhancing FUS targeting of the NAc. This power setting corresponds to a FUS pressure of 7 MPa, in a US gel phantom (measured with a hydrophone). However, based on previous studies,^12^, ^33^ the *in situ* intensity in rats is estimated to be about 50% of this value, on average, due to attenuation through the rat skull. One limitation of the displacement‐versus‐power analysis presented in Figure 3B,C is that the data were acquired from a single animal, with only one ARFI displacement measurement per FUS power level. While the results demonstrate the expected linear trend of increasing displacement with higher power, no statistical measures—such as SD or error bars—could be computed for these plots, as was done in other studies.^32^

The rats used in this study ranged in age from 16 to 31 wk, with their body weight varying from 215 to 565 g. This wide range in weight may have introduced some variation in skull thickness and potentially affected the transmission of FUS energy. Previous work investigated how FUS transmission through the rat skull is influenced by body mass, frequency, and skull location.^33^ Accordingly, skull thickness increases with body weight and the acoustic transmission factor decreases linearly with increasing skull thickness. Specifically, for rats weighing between 90 g and 520 g, skull thickness varied from approximately 0.2–0.6 mm, and the transmission factor ranged from 0.85 to 0.35. Since the weight range in our study was comparable, we estimate that the transmission factors in our cohort to range between approximately 0.35 and 0.65. This considerable variability in US transmission across animals most likely contributed to inter‐subject differences in the FUS pressure delivered to focal region and the measured ARFI displacement.

Another source of variability in the observed ARFI displacements across animals could be the electronic beam steering used to shift the US focus toward the region of interest (e.g., the NAc) during the experiments. Steering the focus away from the natural geometrical focus of the transducer is known to introduce distortions in focal shape and can reduce the peak pressure at the target, depending on the steering depth and angle. These distortions are further compounded by inter‐subject differences in skull geometry and acoustic properties, leading to unpredictable variations in the acoustic field. While numerical simulations can help anticipate these variations, their accuracy is often limited by the complexity of the skull. Hence real‐time displacement feedback provided by ARFI can be leveraged to ensure that the intended dose is accurately delivered and the region of interest is precisely targeted.

The low variability in repeated ARFI measurements at the same site (CoV 9.0% without repositioning) highlights the reproducibility and reliability of MR‐ARFI for measuring displacements induced by FUS. This high reproducibility is essential for minimizing the error in the FUS pressure estimated using MR‐ARFI. However, for this subset of animals, sonication was performed immediately after euthanasia by isoflurane overdose within the MRI suite. These animals were part of a separate protocol and were sacrificed for another study, since at that time, no approved protocol existed for conducting MR‐guided FUS in live, anesthetized animals. The freshly euthanized animals were handled identically to those under anesthesia in terms of positioning, acoustic coupling, MR imaging, and FUS targeting, with the only difference being the absence of anesthesia. However, the lack of physiological motion in these animals may have contributed to the reduced intra‐site variability observed in ARFI displacement measurements.

Although this work demonstrated that MR‐ARFI can estimate intra‐site displacement with high reproducibility, validating the absolute magnitude of the microscopic displacement remains challenging due to its small scale and the absence of a practical, co‐registered external measurement method. Theoretical simulations currently represent the most feasible approach for estimating expected displacement values, but they rely on accurate knowledge of tissue mechanical properties and skull characteristics.

Our findings reveal marked variability in ARFI displacement measurements across different subjects, with an inter‐subject CoV of 43.3% and over three‐fold differences in displacements across experiments. This high variability suggests that even with consistent and specified FUS power settings, factors such as skull geometry, density, and transducer placement, including differences in incident angle on the skull and the degree of electronic steering, may cause deviations in the pressure delivered to the target region. This variability could lead to inconsistent neuromodulation effects, highlighting the need for real‐time monitoring and adjustment of FUS parameters. Since the displacement from ARFI linearly predicts the power deposited, as demonstrated in this work and in other studies,^18^, ^32^, ^34^ ARFI can provide real‐time feedback that allows for estimating and adjusting the FUS dosage during neuromodulation sessions.^30^ A limitation of this study is the absence of acoustic simulations to isolate the influence of specific anatomical and setup parameters (e.g., skull thickness, shape, incident angle, and steering configuration). Incorporating such simulations in future work would allow for a more detailed understanding of how each factor contributes to the observed variability in ARFI measurements.

Despite the large intra‐subject variability noted above, ARFI displacements at the left and right NAc within the animals were correlated. This is likely due to the symmetry between these two targeting cases. In addition, variations in skull density are expected to be lower within the same subject compared to skull density variations across different subjects.

Approximately one‐fifth of the initial MR‐guided FUS targets required secondary adjustments using MR‐ARFI. It is likely that the inherent complexities of skull anatomy can cause substantial aberrations in the location and shape of the FUS focal point. Hence, our findings emphasize the limitations of MR guidance alone and the necessity of incorporating MR‐ARFI into the workflow to enhance targeting precision in MR guided‐FUS treatments.

A common approach to correct the deviations in FUS beam uses simulations on preoperative skull images to find optimal sonication parameters, i.e., phases and optionally amplitudes of US transducer elements. The skull images are typically obtained from CT images^35^, ^36^, ^37^, ^38^ and recently also from MR images.^39^, ^40^, ^41^ This approach is attractive since it is non‐invasive and is employed in some modern transcranial FUS systems. However, due to high complexity of acoustic wave propagation, especially in heterogeneous anatomical structures, this approach might still benefit from closed loop feedback by incorporating the information from MR‐ARFI during FUS procedures.

The MR thermometry sequence demonstrated that the increase in temperature during the ARFI sonication scheme remained within 1°C, even at a power level as high as 22.4 W, which exceeds the 16 W power typically used in our ARFI experiments. Hence, heating during MR‐ARFI acquisitions remains within acceptable limits. A limitation of this study is that the MR thermometry sequence was performed during a sonication scheme using the same parameters as MR‐ARFI, but was not interleaved with the actual MR‐ARFI acquisition. Interleaving MR thermometry with MR‐ARFI, as described in prior studies,^34^, ^42^ would allow for simultaneous measurement of both temperature rise and displacement induced by the acoustic radiation force.

While the spin‐echo‐based ARFI sequence used in this study offers robustness and high SNR, it is relatively slow and requires high number of sonication pulses. Acquisition schemes like segmented EPI acquisition^23^, ^43^ and spiral^25^ imaging can provide a faster alternative at the expense of SNR. Another limitation in this study is that we assumed the elastic tissue properties and tissue boundary conditions remained fairly consistent across animal experiments to infer variation in the delivered pressure from the displacement variation. Future studies could explore integrating MR‐ARFI with preoperative skull imaging simulations to calculate intensity fields.

Human subjects exhibit significant intra‐ and inter‐subject variability in both skull thickness and skull density ratio.^44^ These anatomical differences, combined with the need to target various or deeper intracranial regions, increase the risk of targeting errors and dose inconsistency. This highlights the importance of incorporating MR‐ARFI–based displacement feedback into clinical workflows to enable real‐time assessment and correction of beam targeting and acoustic dose delivery. We are currently working on implementing ARFI in clinical MRI systems to evaluate its utility in clinical practice.

In conclusion, our study using MR‐ARFI demonstrates very large variations (over three‐fold) in the FUS‐induced displacements at the target region and consequently the delivered FUS pressure across animals. Furthermore, FUS targeting errors occurred in one of five animals, even when initial MR‐guidance was used. Hence, MR‐ARFI can improve the accuracy of FUS targeting and provide a reliable method for estimating and adjusting the US pressure delivered during neuromodulation procedures. By reducing the variability in FUS dosage delivery and targeting errors during transcranial FUS procedures, MR‐ARFI promises more consistent and effective treatment outcomes, ultimately enhancing the clinical utility of MRI‐guided FUS in neuromodulation and other brain therapies.
